# Ethnic Composition of Couples and Mutual Health Benefit Receipt: Register-Based Evidence from Finland

**DOI:** 10.3390/ijerph19010073

**Published:** 2021-12-22

**Authors:** Jan Saarela, Maria Stanfors, Mikael Rostila

**Affiliations:** 1 Demography Unit, Strandgatan 2, Åbo Akademi University, 65100 Vasa, Finland; 2 Department of Economic History, Lund University, 22007 Lund, Sweden; maria.stanfors@ehl.lu.se; 3 Department of Public Health Sciences, Stockholm University, 10691 Stockholm, Sweden; mikael.rostila@su.se

**Keywords:** health benefit receipt, couples, ethnicity, intermarriage

## Abstract

The literature on health dependencies among partners typically ignores diversity of partnership characteristics. One salient example is the ethnic composition. We extend prior work on partnerships and health by investigating how married and cohabiting partners mutually influence each other’s receipt of health-related benefits, focusing on how such correlations vary with the couple’s ethnic composition. We study partners’ mutual receipt of sickness allowance and disability pension in ethnically endogamous and exogamous couples in Finland. The population consists of native individuals in similar socioeconomic positions but belonging to two different ethnic groups—Finnish and Swedish speakers—who differ in health and family life. Using data from population registers, we estimate discrete-time hazard models for first-time benefit receipt, as related to partner’s benefit receipt, among midlife couples. We found evidence of mutual receipt of health benefits in both endogamous and exogamous couples, the correlation being strongest for disability pension. Partner correlation in disability pension receipt is slightly stronger in endogamous Swedish than in endogamous Finnish couples, while women in exogamous couples are slightly less sensitive to men’s receipt than vice versa. The results show that mutual health may be heterogeneous across couples that differ in ethnic composition.

## 1. Introduction

Many studies have documented that partners, respectively, affect each other’s health [1,2,3,4,5,6,7,8]. Partnered individuals, and married people in particular, are generally healthier and live longer than others, which is especially true for men. Health benefits may accumulate due to protective effects that enable income pooling and increase economic resources [9], which in turn provide emotional support, social networks, and social control [10,11]. Likewise, while partners may influence the uptake of health-related benefits and improve quality of life, they may also affect health behaviors negatively, in terms of smoking, heavy drinking, poor diet, and inadequate exercise [12,13,14,15]. Among the elderly, hospitalization or death have been found to increase the mortality and morbidity risks of the spouse [16,17]. Mutual relations of this kind confirm that one of the many functions of partnership is health production, implying that the connectedness of individuals should be considered when studying health outcomes [18,19,20].

The literature on health dependencies among partners typically ignores diversity when it comes to partnership characteristics, and thus obscures effect heterogeneity [21]. Different combinations of partnership characteristics, whether they are demographic or social, have consequences on many facets of life and may be important for the health of both parties. One salient example is the ethnic or racial composition of the couple. To the extent that partnerships across ethnic or racial borders have fewer resources, are not fully accepted socially, or are subject to discrimination, such partnerships may be adversely impacted in their health due to stress [22]. Interethnic and interracial partnerships have increased considerably in number in both the U.S. and Europe, with extant studies focusing on health, but few have investigated associations between partnership composition and health. Most of the literature has been concerned with how one partner’s ethnicity or race affects and dominates health within the couple [23,24]. The empirical evidence on the role of the ethnic composition itself, which we are concerned with in this paper, has been less studied and is inconclusive [25].

We extend prior work on partnerships and health by investigating how married and cohabiting partners mutually influence each other’s receipt of health-related benefits. The primary aim is to analyze how such correlations vary by the couple’s ethnic composition. Using longitudinal population register data from Finland, we study a person’s first-time receipt of sickness allowance or disability pension as a function of the partner’s receipt of the same. Both benefits are related to reduced working capacity before statutory retirement age, and they are by far the two most common health-related benefits available [26]. These two measures could reflect not only health dependencies between partners but also rational decisions concerning mutual leisure time vs. economic loss within the partnership.

Finland provides an interesting case study that contrasts the dominant U.S. literature on interethnic and interracial partnerships and health. The country is as a comprehensive welfare state with universal coverage of social and medical services, high uptake of sickness allowance and disability pensions, modest social disparities and income inequality, a well-projected ageing process, and high levels of gender equality. Finland also features two ethnic groups—Finnish and Swedish speakers—which are distinguished in the population register by their unique mother tongue. Finnish speakers amount to 90 percent of the total population, and Swedish speakers make up five percent. Both groups are native and have a similar socioeconomic position, but Swedish speakers are notably healthier than Finnish speakers on objective health measures, including sickness allowance, disability pensions, and life expectancy [27]. Although intermarriage has been high during the past decades, the two groups are distinct with regard to practices regarding family life and stability of the nuclear family [28]. The most illustrative evidence is perhaps that both divorce rates and separation rates from non-marital unions are higher among Swedish than Finnish speakers, and higher in mixed unions as compared with endogamous Finnish unions [29]. No exhaustive explanation has been provided to the Swedish–Finnish health gradient, nor to the difference in separation and divorce rates. Finland has experienced foreign-born immigration only very recently. In the cohorts studied here, intermarriage across ethnic lines other than the Swedish and Finnish lines has therefore been rare, and it also lies beyond the scope of this paper. By focusing on the ethnolinguistic composition of these native couples, we contribute to the small but growing literature that addresses heterogeneity in the health impacts of partnerships.

## 2. Theoretical Underpinnings

### 2.1. Partnership and Health

Much of the literature on partnered individuals’ health have emphasized the protective importance of the social structures in which individuals live their lives [10,30]. A greater sense of connectedness through the partnership may foster a healthy lifestyle [7,31]. Marriage and other close social relationships may offer support as a buffer against various stressors that could be damaging to mental and physical health [11]. The marital resource model further proposes that marriage itself can improve individuals’ health through access to health-protective resources, such as income pooling [7,9,31]. Another explanation is selection, in that healthy people are more likely to enter partnership and marriage and less likely to divorce [3,32]. A partner’s health is consequently often like that of the other, and these similarities tend to converge over time [33]. 

Theories that integrate marriage markets and health capital formation argue that inter-spousal correlation in health status follows on assortative mating, common health behaviors, shared environmental risk factors, and the direct effects of the health of one spouse on that of the other [34]. Assortative mating means that partners sort on certain characteristics and tend to be similar when it comes to demographic characteristics, preferences, and health-related behavior [14,33]. Heterogamy would then imply less health concordance. In accordance, marital resource theory argues that inter-spousal correlation in health is a function of shared life events and resources, for both good and bad. Thus, the presence of a partner is not necessarily protective, and partners may influence each other negatively in terms of health behavior, and thus contribute to mutual health benefit receipt [12].

Among the elderly, it is commonly found that the hospitalization or death of one spouse affects the other through increased mortality or morbidity risks, and these associations are both immediate and long-term [16,17,35]. Among aging adults, partners can influence each other’s quality of life and health behaviors, such as smoking, drinking, diet, and exercise [13,14]. The partner can also influence the disease risk faced by the other, in terms of diabetes, metabolic syndrome, hypertension, cancer, and depression [33,36,37,38]. There also seem to be strong and long-term interrelations among partners in receipt of sickness allowance and disability pension [15], which could be explained not only by collateral health and informal care needs, but also by potential strategic decisions concerning mutual leisure time vs. income loss. 

Mutual health benefit receipt may consequently also be understood from the perspective of coordinated behaviors, in terms of how economists model labor supply decisions. Individuals are then supposed to strike an optimal balance between the cost of foregone leisure and the benefits of increased income through paid employment [39,40]. Depending on their characteristics, coupled persons may pool resources and influence each other’s decisions [41,42]. The quality of the relationship will presumably have a strong impact on this mutual decision. The expectation of spending more time together following sickness absence or disability retirement is most likely more prevalent among couples who have established joint activities and who enjoy a high-quality relationship. From this perspective, partners in a stable and high-quality relationship would be likely to strive towards joint decisions on health benefit uptake. Partners whose relationships are strained and unstable, on the other hand, may be less disposed toward such mutual actions.

### 2.2. Ethnic Composition of Couples, Health, and Mutual Health Benefit Receipt

Financial and psychosocial resources that improve health and protect well-being are known to differ across population subgroups [9,43,44], and such differentials may impact health-related decisions concerning the receipt of health benefits. The extended kin networks and social integration that a partner brings to a relationship may improve the couple’s health and well-being [4,7,31], though this may be more limited among heterogamous couples than those that are homogamous [45]. Economic resources and wealth may also influence decisions related to the receipt of sickness benefits. Incentives derive not only from one own’s economic resources and life course experiences, but also from those of the spouse. The level of economic resources within ethnic groups may therefore contribute to any variation in mutual health benefit uptake across exogamous and endogamous couples.

The limited research, primarily from the U.S., on how the ethnic or racial composition of couples affects health, has mainly hypothesized that exogamous partnerships would be less health-protective than endogamous ones, because of less efficient communication and coordination within the couple and fewer social resources [11,46]. Exogamy may also be less health-protective because of stress experiences that result in psychological and physical health problems. This would be the case if the mixed partnership is not fully socially accepted or is subject to discrimination [30,47]. Empirical evidence on this matter is inconclusive, however [22,23,24,25]. A given partner’s race appears to dominate the mere partner composition in this regard, and it typically distinguishes between White people and non-White people. Contrary to the arguments of stress process theory, having a White spouse is associated with better self-rated health for non-White people, while intermarried White people tend to have worse health than those in non-mixed marriages [24]. This would suggest that the greater resources brought by a majority White partner to the relationship may go so far as to benefit their minority partner’s health. Partners of different race or ethnicity may thus bring different economic and psychosocial resources to the partnership [11,44,48].

Gender may moderate the associations between relationship-related resources, stress, and health. Overall, being married seems to matter more for men’s health than for women’s health. This is in line with the argument about gendered specialization of household labor [49], where women do more unpaid work, including caregiving; though the extent of specialization may differ across ethnic or racial groups [25]. Thus, the health impact of a given partner’s ethnicity or race may matter more for men than for women. On the other hand, women generally experience more stress than men, which may explain why White women in interracial partnerships in the U.S. experience more psychological distress [22]. In that case, the health impact of the partnership’s mixed composition may matter more for women than for men.

### 2.3. Study Contribution

We investigated partners’ mutual receipt of sickness allowance and disability pension in Finland in the period 1987–2011 among native-born couples with different ethnic characteristics. We distinguish between endogamous Finnish couples, endogamous Swedish couples, exogamous couples with a Finnish-speaking man and Swedish-speaking woman, and exogamous couples with a Swedish-speaking man and Finnish-speaking woman. The ethnic composition of the couple should be considered as a proxy for the partnership context of relevance for health concordance, rather than as a causal factor.

As in most other countries, endogamy is the prevailing norm for mate selection in Finland, though intermarriage has become more common, particularly in the case of Swedish speakers. In the 1950s, approximately 20 percent of the Swedish speakers married a Finnish speaker. This share rose until the 1980s when it leveled off, and today about 40 percent of Swedish speakers form a union with a Finnish speaker [50]. Unlike intermarriage among many other ethnic or racial groups, there are few easily discernable differences between Finnish and Swedish speakers except for their mother tongue, which is a marker for ethnic affiliation in the population register. Discrimination against and social stigma associated with this form of exogamy is therefore very limited. There are nevertheless group differences of relevance, in that Swedish speakers have better health and live longer than Finnish speakers [27,50]. It has been argued that these differences in health may be partly associated with Swedish speakers having better and more extensive social networks, as they form a minority group who live, geographically, relatively concentrated [28]; nevertheless, no exhaustive explanation has been provided. There are also ethnic group differences in practices related to family life and the stability of the nuclear family, which presumably reflect differences in relationship quality [51,52]. An illustrative example is that Finnish speakers have almost twice as high a risk of divorce and separation from their cohabiting partner compared with Swedish speakers. In addition, exogamous unions have an elevated, or approximately ten percent higher, risk of ending in divorce or separation compared with endogamous Finnish unions [29]. As already noted, Finland has experienced foreign-born immigration only very recently. In the cohorts studied, intermarriage across ethnic lines other than the Swedish and Finnish has therefore been rare and lies beyond the scope of this paper. 

Based on this context and the mechanisms involved, we make the following conjectures. Firstly, considering that all the study individuals are part of the same institutional setting and health benefits system, we expect to find evidence for partners’ mutual receipt of health benefits in both endogamous and exogamous couples. Secondly, irrespective of the couple’s ethnic composition, we expect that partner concordance in benefit receipt would be stronger for disability pension than for sickness allowance, because the former indicates a more severe state of poor health. It also reflects a permanent exit from the labor market and could thereby be influenced to a greater extent by joint retirement decisions between partners. Thirdly, we expect stronger cross-spousal dependence in health and benefit receipt in endogamous Swedish couples than in endogamous Finnish couples. This is because greater union stability and presumed higher marital satisfaction in the Swedish couples suggest more commitment, stronger family ties, greater interdependence with regard to health, and more efficient communication and coordination. Fourth, because women in general are more likely to shoulder more caregiving responsibilities and suffer from related stress reactions, we expect the woman to be more sensitive to the man’s benefit receipt than vice versa. Fifth, if the uptake behavior that is typical for an ethnic group dominates any gender-specific sensitivity to a partner’s benefit receipt, we expect that, in an exogamous couple, the man will be more sensitive to the woman’s benefit receipt than the woman to the man’s receipt.

## 3. Materials and Methods

### 3.1. Data Source

We used a large longitudinal dataset of married or cohabiting individuals in Finland. The data cover the period 1987–2011, came from various administrative records maintained by Statistics Finland, and were used with permission number TK-53-768-12. They constitute two similarly constructed samples: a 5 percent random sample of Finnish speakers and a 20 percent random sample of Swedish speakers. The sample of Swedish speakers is larger because they amount to only about 5 percent of the total population of the country. Based on each individual’s unique ethnic affiliation in the population register, we could distinguish between endogamous Finnish-speaking couples, endogamous Swedish-speaking couples, exogamous couples with a Finnish-speaking man and a Swedish-speaking woman, and exogamous couples with a Swedish-speaking man and a Finnish-speaking woman.

The data consist of men and women in heterosexual partnerships, in which both partners were native-born Finnish or Swedish speakers and 40–54 years old at entry into the study in 1992. In these ages, disability retirement in terms of receiving disability pension is in practice the only form of retirement. Individuals were followed over time, for 20 years at most or until they turned 65 years old. We consequently studied midlife individuals who were past the core family-formation years and eligible for both sickness allowance and disability pension. People with recent health problems, i.e., those who had received any sickness allowance or disability pension in the five-year period before 1992, were excluded from analysis. Individuals were right-censored when either they or their partner turned 65, at divorce or separation, or the emigration or death of either partner. The analytical sample contained 36,034 couples with individuals born in 1937–1961. In total, there were 22,348 sickness allowance recipients and 7807 disability pension recipients.

### 3.2. Outcome Measures

The outcome variables of interest were receipt of sickness allowance and receipt of disability pension. The Social Insurance Institution of Finland (KELA) pays sickness allowance to non-retired residents aged 16–67 years in case of work incapacity due to illness. A statement from a practitioner in medicine is a precondition for the benefit. The sickness allowance is available after a specified waiting period, which is the first day of illness and the subsequent nine working days. The full benefit can be received for a maximum period of approximately 1 calendar year (300 working days), within 2 years. The amount of compensation depends on previous earnings and other benefits. The maximum level of disbursement is 70 percent, up to an upper limit, after which it decreases. If work incapacity persists after sick leave, one may apply for disability pension, which generally means a permanent withdrawal from the labor market. While sickness allowance receipt reflects temporary illness, receipt of disability pension indicates permanent illness or long-term health problems to an extent that prevents the individual from working. The proportion of disability pensioners in Finland increases notably after age 50 years, while the proportion of sickness allowance recipients drops after age 55 years [27].

Thus, both health benefits are related to reduced working capacity before statutory retirement age and are conditional on a diagnosis statement issued by a practitioner in medicine. Each benefit may mark the onset of severe health problems for some individuals, but overall, they reflect less serious health conditions than hospitalization or death. For each calendar year, we know if a person received any sickness allowance and if he or she received disability pension. There is no information about the medical reason for sickness allowance or disability pension in the data.

### 3.3. Analytic Strategy

The probability of benefit receipt was estimated with discrete-time hazard models with time-varying control variables, which is a common approach for analyses of this kind [15]. In the models, time is discrete because of the calendar-wise structure of the data. The data were consequently episode-split by each calendar year, and parameters were estimated on a yearly basis. Since all information was at the calendar year level, same-year occurrences, i.e., receipt during the same calendar year for both partners, could not be sequenced.

The main explanatory variable was the time since a partner’s first receipt of sickness allowance or disability pension, and the dependent variable was the ego’s first receipt of sickness allowance or disability pension. Thus, we identified the first time a person (the ego) received sickness allowance or disability pension and related this to receipt by the partner. Our aim was to evaluate how a person’s receipt of a benefit depends on the benefit receipt of the partner, and in particular, to compare this estimate across couples who differ on ethnic composition. To avoid statistical complications from potential inter-spousal dependence, we estimated models separately for men and women. For the same couples, therefore, there are separate models where either the man or the woman is the study person (the ego). All analyses were carried out with SPSS 24.

Several control variables were included for the study person, the partner, and the couple. They have previously been shown to be relevant for analyses of this kind [15]. For the individual and the partner, we used age, educational level, educational field, labor market status, and income quintile. For the couple, we controlled for the age difference between partners, marital status, union duration, presence of children in the household, housing tenure, region of residence, and the municipality’s degree of urbanization. We also controlled for observation year. All variables were time-varying on a yearly basis, except educational level, educational field, and the age difference between the partners.

## 4. Results

Table 1 summarizes the descriptive statistics of the data by sex of the ego and ethnic composition of the couple. The category ‘Finnish men with Finnish women’ means that the study persons are Finnish-speaking men and their partners are Finnish-speaking women, while ‘Finnish women with Finnish men’ means that the study persons are Finnish-speaking women and their partners are Finnish-speaking men. Because the data concern couples, the variable distributions for men and women within ethnic configurations mirror each other.

Endogamous Finnish couples, endogamous Swedish couples, and exogamous couples do not differ much regarding demographic and socioeconomic characteristics, but do differ, predominantly, regarding where they live. The differences in region of residence reflect that almost all Swedish speakers in Finland live on the southern and western coastlines of the country, and within this area, exogamous unions are most common in the south.

Table 1 also shows that there are notable differences in receipt of sickness allowance and disability pension by ethnic composition of the couples. For both men and women, Finnish speakers in endogamous couples have higher shares of sickness allowance and disability pension receipt than Swedish speakers in endogamous couples. Furthermore, Finnish speakers in exogamous couples have lower shares of sickness allowance and disability pension than Finnish speakers in endogamous Finnish couples. Similarly, Swedish speakers in exogamous couples have lower shares of sickness allowance and disability pension than have Swedish speakers in endogamous Swedish couples.

Table 2 summarizes the results for models in which we established the main explanatory variable by merging the categories for time since partner’s benefit receipt into one category that includes the same and subsequent calendar years. Consequently, the estimates display the study person’s risk of benefit receipt in the same or subsequent years as the partner received the benefit for the first time, as compared with the situation if the partner had not received the benefit (which is set to 1). Each estimate comes from a separate regression. The first column (SA vs. SA) refers to the study person’s risk of sickness allowance receipt as related to their partner’s receipt of sickness allowance. The second column (DP vs. DP) is the study person’s risk of disability pension as related to their partner’s receipt of disability pension. The third (SA vs. DP) and fourth (DP vs. SA) columns refer to cross-dependency in terms of benefit types. The upper panel gives unadjusted estimates, and the lower panel provides estimates for models in which all control variables have been included. The taxonomy regarding the ethnic composition of the couple is the same as in the previous table. Thus, the first two rows refer to endogamous Finnish couples, where the first row shows the findings for models in which the man is the study person, and the second row shows those for models in which the woman is the study person. The third and fourth rows show the findings for endogamous Swedish couples, the fifth and sixth rows show the findings for exogamous couples with a Finnish man and a Swedish woman, and the seventh and eighth rows show the findings for exogamous couples with a Swedish man and a Finnish woman.

We found ample evidence for partners’ mutual benefit receipt. If the partner has received a benefit, the study person has a notably increased risk of receiving the same and, to some extent, even the other type of benefit. Some, but far from all, of these associations relate to socioeconomic and demographic factors, as the estimates also remain considerable when we include the control variables. Associations are stronger for the way in which disability pension receipt relates to partner’s disability pension receipt, as compared with the way in which sickness allowance receipt relates to partner’s sickness allowance receipt. Patterns for cross-dependency regarding benefit types are somewhat less clear, although in most instances there are positive associations. For instance, for Finnish men who live with Finnish women, the risk of receiving sickness allowance is raised by a factor of 1.28 (95% CI: 1.21–1.35) if the partner has received sickness allowance, as compared with if the partner has not. For disability pension, the risk is raised by a factor of 1.71 (95% CI: 1.49–1.96). If the partner has received a disability pension, the sickness allowance risk is raised by a factor of 1.32 (95% CI: 1.17–1.48). If the partner has received sickness allowance, the disability pension risk is raised by a factor of 1.37 (95% CI: 1.26–1.48).

In endogamous Swedish couples, estimates for how sickness allowance receipt relates to partner’s sickness allowance receipt are the same as in endogamous Finnish couples. Estimates for how disability pension receipt relates to partner’s disability pension receipt, on the other hand, are higher in endogamous Swedish couples than in endogamous Finnish couples, or 2.53 (95% CI: 1.78–3.59) for men and 2.65 (95% CI: 1.87–3.74) for women, although the confidence intervals overlap. In exogamous couples with a Swedish man and a Finnish woman, the estimates are even larger, or around 1.6 for sickness allowance receipt and 3.0–3.5 for disability pension receipt, albeit the confidence intervals are wide. In the other type of exogamous couple, with a Finnish man and a Swedish woman, the estimates are smaller in size and border on being statistically significant because of the small group size.

In both endogamous Finnish couples and endogamous Swedish couples, women tend to be slightly more sensitive to their partner’s benefit receipt than vice versa. A Finnish woman’s risk of disability pension receipt is raised by a factor of 2.07 if her Finnish partner (the man) has received disability pension, while a Finnish man’s risk of disability pension receipt is raised by a factor of 1.71 if his Finnish partner (the woman) has received disability pension. A similar but less distinct difference in the size of the estimates can be seen for endogamous Swedish couples (2.65 vs. 2.53).

In exogamous couples, the pattern is rather the opposite. The disability pension risk for a Swedish man in an exogamous couple is raised by a factor of 3.54 if the (Finnish) woman has received a disability pension, while that for a Finnish woman increases by a factor of 3.01 if the (Swedish) man has received a disability pension. There is a similar, but less distinct, pattern for exogamous couples consisting of Finnish men and Swedish women. Confidence intervals are nevertheless too wide to facilitate any rigorous conclusion on this point.

Because all data are on a calendar year basis, we cannot sequence partners’ mutual benefit receipt if it occurred during the same calendar year. To study whether the results discussed above are sensitive to this impediment, we performed parallel analyses in which same-year occurrences were excluded, and where we thus focused on the study person’s risk of benefit receipt one or more calendar years after partner’s benefit receipt. The results are summarized in Table 3, showing that the overall conclusions based on these estimates do not differ considerably from those reported above.

## 5. Discussion

Using register-based data that cover the years 1987–2011, we have studied partners’ mutual receipt of sickness allowance and disability pension in Finland. We have assessed behaviors in two ethnic groups—Finnish speakers and Swedish speakers. Both are native with a similar socioeconomic position, but Swedish speakers are generally healthier and have lower divorce and separation rates. We have tested for potential differences in partnership resources across ethnic groups in a context where discrimination against intermarried couples should be minor, especially in relation to what has been observed for intermarried couples in the United States. The present study has contributed to the small but growing literature that addresses heterogeneity in the health implications of partnerships. We have also contributed to the research area by extending health outcomes to less severe states than mortality and morbidity, which have been studied before, although almost exclusively for the United States. Additionally, we have extended the study area of partners’ mutual receipt of benefits and heterogeneity in the health impacts of partnership to a context that features an egalitarian and generous welfare state, in which the receipt of health benefits is generally high.

Discrete-time hazard models were estimated for individuals aged 40–65 years, with emphasis on behaviors typical for couples with a different ethnic composition. In line with expectations, we found ample evidence for partners’ mutual benefit receipt in both endogamous and exogamous couples (Hypothesis 1). These findings could be explained by mutual health influence between partners, as the state of health for each was often similar and tended to converge over time [33]. Such inter-spousal correlation in health status may follow on assortative mating, common health behaviors, shared environmental risk factors, and direct effects of the health of one spouse on the health of the other [12]. Thus, the presence of a partner is not necessarily protective, and partners may influence each other negatively, which in turn may contribute to mutual health benefit receipt, as found here. However, if sick leave or disability pension can be received irrespective of health status, which we cannot observe explicitly, these findings may also be interpreted using economic theories, suggesting that partners strike an optimal balance between the cost of foregone leisure and the benefits of increased income through paid employment [39,40]. If married or cohabiting couples place great value on leisure time spent together, they might consequently mutually withdraw from the labor market. The stronger correlation we found for disability pension than for sickness allowance (Hypothesis 2) may relate to the fact that it reflects a permanent exit from the labor market and is thereby more strongly influenced by joint retirement decisions. Yet, it may equally well relate to the fact that disability pension, compared with sickness allowance, generally reflects a poorer health situation, and that poorer health may have a stronger impact on partner’s health than better health.

Previous research has suggested that relationship quality and marital satisfaction will strongly impact retirement decisions [41,42]. In line with these findings, we could see that endogamous Swedish couples, who can be assumed to have higher relationship quality than Finnish couples, displayed slightly stronger associations with regard to mutual health benefit receipt (Hypothesis 3). Higher satisfaction with the relationship would then potentially be reflected in stronger collateral health. An alternative explanation would be that, if the relationship quality is higher in endogamous Swedish couples in the absence of divorce or separation as well, they may be more likely than endogamous Finnish couples to look forward to opportunities for more mutual leisure time. Given that their decisions can be made irrespective of health status, their uptake of health benefits may therefore be stronger correlated. The lower concordance in benefit receipt among endogamous Finnish couples would then reflect the mirror image, i.e., that lower relationship quality may enforce continued participation in the labor force as an escape from relationship tensions. However, we cannot study the latent mechanisms and cannot therefore empirically distinguish the factors at play.

In both endogamous Swedish couples and endogamous Finnish couples, women tend to be more sensitive to the man’s benefit receipt than vice versa (Hypothesis 4). This is presumably because women are more likely than men to shoulder caregiving responsibilities and suffer from resulting stress reactions, especially within this context of a comprehensive welfare state with fairly egalitarian gender roles. For exogamous couples, on the other hand, we found some support for the argument that behaviors typical for an ethnic group dominate the gender-specific sensitivity to partner’s benefit receipt (Hypothesis 5). Thus, in exogamous couples, men’s response to women’s uptake of disability pension is somewhat stronger than women’s response to men’s uptake of the same benefit. However, this could also be a reflection of selection on some unobservable traits, meaning that partners who form an exogamous couple, and particularly those consisting of a Swedish man and Finnish woman, are inherently different from others.

The high-quality Finnish register data we have used display several strengths when compared with other sources of data, as we need not be concerned with issues related to non-response, selective participation, or attrition. However, there are notable limitations as well. We lacked information about the medical diagnoses or specific health problems that could underlie the mutual receipt of benefits. Data on the length of sickness spells were also crude. Being a register-based study, we could not observe the norms, the preferences, nor the other behaviors in an explicit manner. It needs to be stressed, therefore, that our data did not allow us to separate the potential mechanisms involved and, therefore, we were unable to separate the pathways behind the correlated benefit receipt in couples. 

## 6. Conclusions

Our findings could be explained both by collateral health effects and by joint decisions within a given couple. Data other than the registered-based records used here would be needed to separate, test, and quantify these two main components of the health benefit correlation we have observed here. Nevertheless, our results clearly cast light on the important issue of mutual health in couples, and particularly on the fact that associations of this kind may be heterogeneous across couples that differ in ethnic composition. Future studies could seek to incorporate theoretical models that articulate the pathways involved and test them with the help of rigorous empirical models. 

## Figures and Tables

**Table 1:** Descriptive statistics of the analytic data.

	Endogamous Finnish-Speaking Couples		Endogamous Swedish-Speaking Couples		Exogamous Couples with Finnish-Speaking Man		Exogamous Couples with Swedish-Speaking Man	
	Finnish Men with Finnish Women	Finnish Women with Finnish Men	Swedish Men with Swedish Women	Swedish Women with Swedish Men	Finnish Men with Swedish Women	Swedish Women with Finnish Men	Swedish Men with Finnish Women	Finnish Women with Swedish Men
Age in years (%)								
40–44	10.2	15.5	8.0	13.4	9.2	16.7	9.7	12.2
45–49	21.8	24.9	19.7	23.7	21.1	24.6	20.5	23.9
50–54	26.8	27.0	27.1	27.5	27.3	27.0	27.0	27.8
55–59	24.7	21.9	26.3	23.6	25.4	21.5	25.3	23.9
60–64	16.5	10.6	18.9	11.8	16.9	10.3	17.5	12.2
Educational level (%)								
Primary	30.3	28.6	34.0	30.5	31.0	32.3	30.7	30.6
Secondary	32.5	36.7	26.5	34.1	28.7	28.0	21.9	30.0
Tertiary	37.2	34.7	39.6	35.5	40.3	39.7	47.4	39.3
Educational field (%)								
Science	52.9	31.3	48.3	29.3	51.7	31.3	53.3	31.7
Welfare	8.3	32.4	7.7	33.7	9.0	28.0	7.3	26.2
General	38.8	36.3	44.0	37.0	39.3	40.7	39.4	42.1
Labour market status (%)								
Employed	77.2	79.3	83.5	83.6	79.5	81.8	80.4	79.8
Unemployed	8.1	8.4	4.1	5.0	6.5	6.3	6.2	7.2
Outside the labour force	14.7	12.2	12.4	11.4	14.0	11.9	13.4	13.0
Income quintile (%)								
First	17.1	23.2	16.3	22.9	15.5	23.3	13.6	21.8
Second	14.8	24.7	13.6	25.0	14.0	22.2	13.5	23.0
Third	13.9	26.1	13.5	26.9	11.5	23.0	11.5	23.2
Fourth	24.2	16.2	22.6	16.1	22.5	18.6	21.3	18.6
Fifth	30.0	9.7	34.0	9.2	36.6	12.9	40.1	13.4
Age difference vs. partner (%)								
At most two years	60.0	60.0	62.0	62.0	58.8	58.8	61.4	61.4
At least three years older	32.5	7.6	32.7	5.2	35.9	5.3	27.7	10.9
At least three years younger	7.6	32.5	5.2	32.7	5.3	35.9	10.9	27.7
Partner’s educational level (%)								
Primary	28.6	30.3	30.5	34.0	32.3	31.0	39.3	30.7
Secondary	36.7	32.5	34.1	26.5	28.0	28.7	30.6	21.9
Tertiary	34.7	37.2	35.5	39.6	39.7	40.3	30.0	47.4
Partner’s educational field (%)								
Science	31.3	52.9	29.3	48.3	31.3	51.7	31.7	53.3
Welfare	32.4	8.3	33.7	7.7	28.0	9.0	26.2	7.3
General	36.3	38.8	37.0	44.0	40.7	39.3	42.1	39.4
Partner’s labour market status (%)								
Employed	79.3	77.2	83.6	83.5	81.8	79.5	79.8	80.4
Unemployed	8.4	8.1	5.0	4.1	6.3	6.5	7.2	6.2
Outside the labour market	12.2	14.7	11.4	12.4	11.9	14.0	13.0	13.4
Partner’s income quintile (%)								
First	23.2	17.1	22.9	16.3	23.3	15.5	21.8	13.6
Second	24.7	14.8	25.0	13.6	22.2	14.0	23.0	13.5
Third	26.1	13.9	26.9	13.5	23.0	11.5	23.2	11.5
Fourth	16.2	24.2	16.1	22.6	18.6	22.5	18.6	21.3
Fifth	9.7	30.0	9.2	34.0	12.9	36.6	13.4	40.1
Marital status (%)								
Married	93.2	93.2	94.4	94.4	90.8	90.8	92.6	92.6
Cohabitants	6.8	6.8	5.6	5.6	9.2	9.2	7.4	7.4
Union duration (%)								
At least five years	94.9	94.9	96.0	96.0	93.4	93.4	94.7	94.7
Less than five years	5.1	5.1	4.0	4.0	6.6	6.6	5.3	5.3
Children in the household (%)								
Yes	51.6	51.6	55.9	55.9	52.6	52.6	49.1	49.1
No	48.4	48.4	44.1	44.1	47.4	47.4	50.9	50.9
Homeowners (%)								
Yes	89.5	89.5	91.3	91.3	85.7	85.7	87.3	87.3
No	10.5	10.5	8.7	8.7	14.3	14.3	12.7	12.7
Region of residence (%)								
Helsinki area	16.7	16.7	14.7	14.7	35.8	35.8	40.6	40.6
Rest of Southern Finland	17.2	17.2	44.8	44.8	36.7	36.7	33.4	33.4
Western Finland	35.4	35.4	40.3	40.3	23.3	23.3	22.6	22.6
Eastern Finland	18.2	18.2	0.1	0.1	3.3	3.3	2.5	2.5
Northern Finland	12.5	12.5	0.1	0.1	0.8	0.8	0.8	0.8
Degree of urbanisation (%)								
Urban	42.1	42.1	31.3	31.3	54.3	54.3	55.9	55.9
Semi-urban	36.0	36.0	33.4	33.4	31.2	31.2	29.8	29.8
Rural	21.9	21.9	35.3	35.3	14.6	14.6	14.3	14.3
Period (%)								
1992–1996	26.4	26.4	27.5	27.5	27.1	27.1	29.0	29.0
1997–2001	29.8	29.8	30.0	30.0	30.0	30.0	30.2	30.2
2002–2006	26.1	26.1	25.8	25.8	25.9	25.9	25.2	25.2
2007–2011	17.7	17.7	16.7	16.7	16.9	16.9	15.7	15.7
# sickness allowance recipients	8810	8730	1654	1584	362	352	388	468
# disability pension recipients	3349	2883	554	477	151	115	135	143
# partners with sickness allowance	8730	8810	1584	1654	352	362	468	388
# partners with disability pension	2883	3349	477	554	115	151	143	135
# individuals	27,630	27,630	5464	5464	1321	1321	1619	1619
# person-years	382,998	382,998	78,153	78,153	17,789	17,789	21,940	21,940

Note: The description is for the complete observation period, i.e., irrespective of the outcome studied. # means ‘number of’. For Educational field, ‘Science’ refers to social sciences, business and law, science, and engineering, manufacturning and construction, ‘Welfare’ to education, health and welfare, and services, and ‘General’ to general programmes, humanities and arts, agriculture, and unknown. Degree of urbanisation is for the municipality of residence, and follows Statistics Finland’s classification.

**Table 2:** Risk of benefit receipt in relation to partner’s receipt, by type of benefit, sex of the study person and ethnolinguistic composition of the household, unadjusted and adjusted estimates.

	SA vs. SA		DP vs. DP		SA vs. DP		DP vs. SA	
Unadjusted estimates								
Finnish men with Finnish women	1.35	(1.29–1.42)	2.27	(2.01–2.56)	1.28	(1.15–1.41)	1.78	(1.65–1.92)
Finnish women with Finnish men	1.42	(1.35–1.49)	2.79	(2.50–3.11)	1.35	(1.23–1.47)	2.00	(1.85–2.16)
Swedish men with Swedish women	1.49	(1.32–1.68)	2.27	(1.64–3.13)	1.28	(1.00–1.65)	1.62	(1.34–1.96)
Swedish women with Swedish men	1.52	(1.35–1.68)	2.63	(1.96–3.52)	1.55	(1.25–1.92)	1.96	(1.61–2.38)
Finnish men with Swedish women	1.35	(1.04–1.75)	2.25	(1.25–4.06)	1.26	(0.76–2.08)	1.40	(0.95–2.06)
Swedish women with Finnish men	1.46	(1.12–1.89)	2.38	(1.36–4.16)	1.12	(0.72–1.76)	1.34	(0.85–2.10)
Swedish men with Finnish women	1.75	(1.38–2.21)	3.48	(2.04–5.96)	1.56	(0.98–2.47)	2.22	(1.54–3.19)
Finnish women with Swedish men	1.74	(1.38–2.19)	2.66	(1.50–4.70)	1.46	(0.94–2.26)	1.75	(1.18–2.60)
Adjusted estimates								
Finnish men with Finnish women	1.28	(1.21–1.35)	1.71	(1.49–1.96)	1.32	(1.17–1.48)	1.37	(1.26–1.48)
Finnish women with Finnish men	1.35	(1.28–1.43)	2.07	(1.82–2.35)	1.40	(1.26–1.56)	1.40	(1.29–1.52)
Swedish men with Swedish women	1.31	(1.16–1.49)	2.53	(1.78–3.59)	1.34	(1.01–1.76)	1.24	(1.01–1.52)
Swedish women with Swedish men	1.32	(1.16–1.50)	2.65	(1.87–3.74)	1.57	(1.22–2.03)	1.56	(1.26–1.93)
Finnish men with Swedish women	1.34	(1.01–1.76)	1.98	(0.96–4.08)	1.26	(0.71–2.21)	1.05	(0.69–1.60)
Swedish women with Finnish men	1.44	(1.08–1.91)	1.88	(0.96–3.67)	0.98	(0.58–1.66)	0.99	(0.60–1.64)
Swedish men with Finnish women	1.57	(1.22–2.01)	3.54	(1.81–6.91)	1.57	(0.92–2.68)	1.92	(1.29–2.87)
Finnish women with Swedish men	1.61	(1.25–2.07)	3.01	(1.53–5.92)	1.75	(1.04–2.94)	1.28	(0.83–1.96)

Note: Estimates (with 95% CIs) refer to study person’s first-time risk of receiving sickness allowance (SA) or disability pension (DP) subsequent to, or during the same calendar year as, partner’s receipt. Situations when the partner has not received the benefit serve as the reference category (set to 1).

**Table 3:** Risk of benefit receipt in relation to partner’s receipt, by type of benefit, sex of the study person and ethnolinguistic composition of the household, unadjusted and adjusted estimates, receipt during same calendar excluded.

	SA vs. SA		DP vs. DP		SA vs. DP		DP vs. SA	
Unadjusted estimates								
Finnish men with Finnish women	1.26	(1.19–1.33)	2.23	(1.95–2.55)	1.26	(1.13–1.41)	1.81	(1.67–1.95)
Finnish women with Finnish men	1.34	(1.26–1.41)	2.85	(2.54–3.20)	1.30	(1.18–1.44)	2.08	(1.92–2.26)
Swedish men with Swedish women	1.40	(1.23–1.59)	2.33	(1.65–3.30)	1.07	(0.79–1.44)	1.65	(1.35–2.02)
Swedish women with Swedish men	1.43	(1.26–1.63)	2.75	(2.02–3.76)	1.59	(1.26–2.01)	1.91	(1.55–2.35)
Finnish men with Swedish women	1.21	(0.91–1.62)	2.02	(1.03–3.97)	1.42	(0.85–2.39)	1.39	(0.92–2.10)
Swedish women with Finnish men	1.33	(1.00–1.77)	2.20	(1.18–4.09)	1.07	(0.65–1.77)	1.46	(0.92–2.31)
Swedish men with Finnish women	1.69	(1.31–2.18)	3.40	(1.88–6.16)	1.32	(0.76–2.29)	2.29	(1.57–3.35)
Finnish women with Swedish men	1.69	(1.32–2.16)	2.43	(1.28–4.63)	1.42	(0.87–2.30)	1.63	(1.06–2.50)
Adjusted estimates								
Finnish men with Finnish women	1.19	(1.12–1.26)	1.67	(1.44–1.95)	1.31	(1.15–1.49)	1.36	(1.25–1.48)
Finnish women with Finnish men	1.27	(1.20–1.35)	2.03	(1.77–2.32)	1.35	(1.20–1.51)	1.42	(1.30–1.55)
Swedish men with Swedish women	1.23	(1.07–1.41)	2.59	(1.78–3.77)	1.09	(0.79–1.51)	1.23	(0.99–1.52)
Swedish women with Swedish men	1.23	(1.07–1.42)	2.61	(1.81–3.75)	1.60	(1.22–2.10)	1.46	(1.16–1.83)
Finnish men with Swedish women	1.20	(0.88–1.64)	1.73	(0.76–3.93)	1.45	(0.80–2.60)	1.03	(0.65–1.61)
Swedish women with Finnish men	1.32	(0.97–1.81)	1.55	(0.75–3.19)	0.94	(0.53–1.68)	1.05	(0.63–1.76)
Swedish men with Finnish women	1.53	(1.17–2.01)	3.40	(1.61–7.19)	1.37	(0.73–2.56)	1.95	(1.28–2.96)
Finnish women with Swedish men	1.56	(1.19–2.06)	2.57	(1.21–5.42)	1.71	(0.97–3.00)	1.12	(0.70–1.79)

Note: Estimates (with 95% CIs) refer to study person’s first-time risk of receiving sickness allowance (SA) or disability pension (DP) subsequent to partner’s receipt, excluding the same calendar year. Situations when the partner has not received the benefit serve as the reference category (set to 1).

## Data Availability

The data were used with Statistics Finland’s permission TK-53-768-12 and can be obtained from Statistics Finland, subject to service fees.
